# The relationship between changes in the korean fertility rate and policies to encourage fertility

**DOI:** 10.1186/s12889-022-14722-4

**Published:** 2022-12-08

**Authors:** Kyuhyoung Jeong, Jiyeon Yoon, Heeran J. Cho, Sunghee Kim, Jihyun Jang

**Affiliations:** 1 Semyung University, Jecheon, South Korea; 2 Seogang University, Seoul, South Korea; 3 Yuhan University, Bucheon, South Korea; 4 Yonsei University, Seoul, South Korea; 5 Pusan National University, Busan, South Korea

**Keywords:** Korean fertility rate, Fertility rate, Decline of fertility rate, Fertility policy, Latent growth model

## Abstract

**Background:**

Korean government has established various policies to counter the low fertility rate since the mid-2000s, but it still has the lowest fertility rate among OECD member countries. This study investigated the relationship between changes in the Korean fertility rate and policies to encourage fertility.

**Methods:**

This study utilized data of the total fertility rate of 250 local governments between 2014 and 2018, and a casebook of local government birth promotion policies. The dependent variable was fertility rate, and the independent variable was fertility promotion policy. Using SPSS 25.0 and M-plus 8.0 programs, descriptive statistical analysis and latent growth modeling were conducted. An unconditional quadratic function change model was selected as a final model of this study.

**Results:**

The average of the initial fertility rate and the linear rate of change in the Korean fertility rate, and the rate of change of the quadratic function were all statistically significant, meaning that the fertility rate of decline increases over time. Also, the linear rate of change and the quadratic function change rate were significant, showing significant differences in the initial level and rate of change of the fertility rate between local governments. Among fertility policies, only the in-kind policy had a significant effect on the initial value of the fertility rate, meaning that the higher the number of in-kind policies, the higher the fertility rate.

**Conclusion:**

This study is crucial as it examined the changes in the fertility rate of Korean local governments as well as the policy factors affecting the fertility rate at a quantitative level.

## Introduction

There is a concern about low fertility in many countries. Since the 2000s, it has been predicted that the low fertility rate would become a global crisis and will reach the level of ultralow fertility within the next 20 years [1]. Each country has planned various policies to solve the problem of low fertility. In most cases, the low fertility policies overlap in various social policy areas covering women, children, family, health, welfare, or labor [2]. Korea has also established numerous policies to counter the low fertility rate at the government level since the mid2000s.

While the fertility rate started to decline before the 1990s in many OECD countries, this issue started in the mid2000s in South Korea, and the fertility rate has declined rapidly [3]. It is discussed that the low fertility rate began in South Korea for several reasons. The labor market trend changed, and more females started to work, but gender inequality is not yet improved [4]. Furthermore, expenditure on childcare and education is getting higher due to competition, and housing prices in metropolitan cities are getting higher [5]. South Korea’s fertility rate is the lowest among OECD member countries, and the rate of decline is the fastest [6]; South Korea is the only country in the OECD countries with a total fertility rate of less than one child [6].

The main factors leading to low fertility in South Korea and background causes are discussed as follows. The first factor is socioeconomic. It is discovered that the fertility rate decreases as women’s education rapidly increases [7, 8]. As women became educated and engaged in economic activities, the marriage period was delayed, or childbirth was abandoned or postponed [9]. The second factor is the rise in housing prices. As housing prices rise, it has become more difficult for unmarried people to marry, and those who are married but do not own housing often give up having children [10]. The third factor is a change in culture and values. In the past, traditional family norms and systems have been maintained in Korea as a Confucian society among Asian countries. However, as the number of single-person, unmarried, and single-person households has been increased in recent years, the fertility rate has also affected. In addition, young people are changing their priorities in life from getting married and having children to their careers and labor [11, 12]. The fourth factor is demographic. As the female population of the major childbearing age group decreased, the marriage rate fell, and the first marriage age arose [13]. These factors can be considered a result of demographic transition. The theory of population change has been discussed in various ways, starting with Notestein. While the traditional society had a high fertility rate and high mortality rate, the modern society has changed to have a low fertility rate and low mortality rate, and this population change trend is expected to continue. In order to overcome the limitations of the classical demographic theory, the second demographic transition theory was proposed. According to the second demographic transition theory of population change, values such as individual freedom or self-actualization are emphasized as society’s values change to post-materialism, and the values change leads to low fertility. The background of the changes can be seen as a result of a wide range of socio-economic changes such as education, economic growth, and the emergence of new roles for women [14, 15].

Low fertility is a critical issue worldwide. Some scholars argue that the result of low fertility is due to liberalism and neoliberalism in common. In other words, the wave of freedom is affecting the quality of life of individuals, and a lot of effort is put into forming and maintaining a family in a capitalist society, making it difficult to form a family [16]. In addition, as society develops and the economy grows, it can be seen that the fertility rate also decreases in inverse proportion [17]. Most countries aim for economic growth as capitalist societies. Accordingly, the economic activity of women, which is the core of childbirth, has been active, and the fertility rate continues to decline [18].

However, most previous studies have dealt with the problem of low fertility in a system, women’s labor force [19–21], and economic activity [22, 23], having a limitation in that they only focused on the role of women in the labor market. In order to solve the problem of low fertility, it is necessary to consider various factors, and it is meaningful to examine the effectiveness of policies. While previous studies mainly suggest policies to counter low fertility, there are only minimal studies that analyze existing policies [24–28]. Furthermore, various research on the low fertility rate primarily examined how the country’s political, social, and cultural atmosphere affects the low fertility rate [1, 24], and they contain a limitation of not examining the effectiveness of practical policies related to low fertility.

South Korea has introduced various policies not only at the central government but also at the local government level to respond to the low fertility problem. The central and local governments are pursuing the direction of the fertility promotion policy by focusing on changes in values and the environment to improve individuals’ quality of life. However, previous studies have not considered low fertility policies comprehensively. They have focused on some areas such as childbirth, childcare, and work-family balance, fragmentarily examining them in relation to low fertility [29].

Various policies have emerged to overcome the low fertility rate and encourage childbirth in South Korea over the past decade. Though “The 4th Basic Plan for Low Fertility and Aging Society (2021–2025)” is implemented three times, the phenomenon of extremely low fertility continues to intensify, and the natural decline of the population and demographic onus (a phenomenon in which economic growth is delayed due to a decrease in the working-age population of ages 15 to 64) has become more severe. Accordingly, the necessity of a new vision arose.

South Korea’s low fertility-related policies can be divided into cash policy, in-kind policy, voucher policy, service policy, and education and public relations policy. As a cash policy, there is a policy to support the cost of treatment for infertile couples and child allowance paid to all children under the age of 7. As an in-kind policy, various policies have been formulated. For example, diapers and formula are provided to low-income families, and iron and folic acid supplements are provided to pregnant women registered to public health centers. Voucher policies include a maternal/newborn health management support project and a coupon issuance policy to support the cost of ultrasound examinations for pregnant women. As for service policy, education and publicity, health-related medical support, childbirth preparation classes, pregnant women’s classes, and various campaigns are being carried out.

However, there are insufficient previous studies examining these policies’ effectiveness in reducing the fertility rate. While some previous studies verified the effectiveness of major policies related to low fertility, most studies were carried out cross-sectionally [30, 31] and had a limitation in that they evaluated the data for a short period of time. This study is meaningful as it utilized longitudinal data to discover whether the fertility promotion policy is effective for low fertility. In this study, we investigated the relationship between changes in the Korean fertility rate and policies encouraging fertility to suggest new policies to counter extremely low fertility.

## Methods

### Data

This study utilized the longitudinal data from 2014 to 2018 on the total fertility rate of 250 local governments (the administration of a town, county, or district) provided by Statistics Korea to estimate changes in the fertility rate. Statistics Korea is the central administrative agency of South Korea, and it is in charge of setting standards for statistics, census, and various statistics. The total fertility rate provided by Statistics Korea is a compilation of birth data reported to each local government in accordance with the Statistics Act and the Act on Family Relations Registration.

### Variables

#### Dependent variable: fertility rate

The fertility rate used in this study is the total fertility rate (TFR). TFR is an index indicating the average number of births that one woman of childbearing age (ages 15–49) is expected to have during her lifetime. It is the sum of fertility rates by age and a representative indicator of the level of fertility.

#### Independent variable: fertility promotion policy

As for the fertility promotion policy, the number of each policy is used by dividing the fertility incentive policies implemented by each local government into cash, in-kind, vouchers, services, education and public relations through the Ministry of Health and Welfare’s 2014 Local Government Birth Encouragement Policy Casebook. In the case of cash, congratulatory cash for childbirth, child support expenses, childcare allowance, treatment expense for infertility couples, postpartum care expenses, and local currency were set, and in-kind items were set as birth celebration gifts, safety kits for infants, nutritional supplements, and diapers. Vouchers were set for the maternal and newborn babysitting business, ultrasound coupons, and fetal malformation test coupons, and services were set for rental of toys and books, use of facilities and programs, helper support, and free medical treatment. Education and publicity were set up as childbirth preparation classes, breastfeeding classes, childcare classes, pregnant women classes, infant health classes, events, festivals, and campaigns.

### Statistical analysis

The analysis method and procedure for solving the research problem are as follows. SPSS 25.0 and M-plus 8.0 programs were used for data handling and model analysis. First, descriptive statistical analysis was conducted to identify the characteristics of major variables. Second, latent growth modeling was conducted to estimate changes in the overall fertility rate and to verify the relationship between fertility policies. To determine the model fit, TLI (Tucker-Lewis Index), CFI (Comparative Fit Index), and RMSEA (Root Mean Square Error of Approximation) were used. The model is suitable if the CFI and TLI are 0.9 or more [32] and the RMSEA is less than 0.1 [33].

### Statistical model

The potential growth model conducted in this study is a model that verifies the longitudinal change flow by identifying the amount of change in the variable over time [34]. The potential growth model consists of a total of two stages. In the first stage, unconditional model analysis is carried out to discover what kind of changes the longitudinal data depict. In step 1, the most suitable model for the data, such as the no-change model, linear change model, and quadratic function change model, is found and explains the characteristics of the initial level and change considering the average and variance of the initial value and rate of change. No-change model is a model that assumes that the fertility rate does not change with time, and the linear change model is a model that assumes that the fertility rate increases or decreases consistently over time. The quadratic function change model is a model that assumes that the rate of change increases or decreases differently over time, like a quadratic curve. The second stage consists of a conditional model analysis that identifies the factors affecting the changing pattern of longitudinal data. In the second stage, the initial value obtained in the first stage and the factors affecting the rate of change are revealed.

## Results

### Descriptive statistics

Descriptive statistical analysis was carried out to confirm the overall characteristics of major variables (Table 1). First, it was found that the fertility rate continued to decrease from 2014 (M = 1.31, SD = 0.26) to 2018 (M = 1.08, SD = 0.25). Looking at the birth incentive policy, cash policies averaged 1.91 (SD = 1.01), followed by 1.41 service policies (SD = 1.62), 1.28 education and promotion policies (SD = 1.93), 0.47 in-kind policies (SD = 0.75), and 0.19 voucher policies (SD = 0.45).

**Table 1:** Descriptive statistics

Classification		Min.	Max.	Mean	S.D.
Fertility Rate	Fertility rate in 2014	0.79	2.43	1.31	0.26
	Fertility rate in 2015	0.81	2.46	1.33	0.26
	Fertility rate in 2016	0.78	2.42	1.27	0.26
	Fertility rate in 2017	0.65	2.10	1.16	0.25
	Fertility rate in 2018	0.60	1.89	1.08	0.25
Birth Promotion Policy	Number of Cash Policies	1	6	1.91	1.01
	Number of In-kind Policies	0	4	0.47	0.75
	Number of Voucher Policies	0	3	0.19	0.45
	Number of Service Polices	0	9	1.41	1.62
	Number of Education and Promotion Policies	0	19	1.28	1.93

### Study model analysis

In this study, the model was analyzed in two stages. In the first stage, the initial value and change rate were estimated through unconditional model analysis, and in the second stage, the relationship between the change in the fertility rate and fertility incentive policies in South Korea was examined based on the initial value and change rate obtained in the first stage through conditional model analysis.

### Analysis of unconditional model

Before proceeding with the conditional model analysis, an unconditional model analysis was performed to understand the change in the fertility rate. In order to identify the optimal change pattern through the unconditional model, the no-change model, the linear change model, and the quadratic function change model were analyzed, respectively (Table 2). The fit of the quadratic function change model for the fertility rate was as follows: CFI and TLI were more than 0.9, and RMSEA was less than 0.1 (χ2 = 107.365, *p* < .001), CFI = 0.949, TLI = 0.915, RMSEA = 0.080. The fit confirms that the quadratic function change model is more suitable for explaining the change in the fertility rate better than the no-change and linear change models. Thus, the quadratic function change model was adopted, and it was confirmed that the rate of change in the fertility rate increased or decreased differently as the quadratic curve over time.

**Table 2:** Model fit of unconditional model

Model	χ2	*df*	CFI	TLI	RMSEA
No-change Model	919.185***	13	0.546	0.651	0.528
Linear Change Model	288.263***	10	0.861	0.861	0.134
Quadratic Function Change Model	107.365***	6	0.949	0.915	0.080

***p < .001

**Fig. 1:**
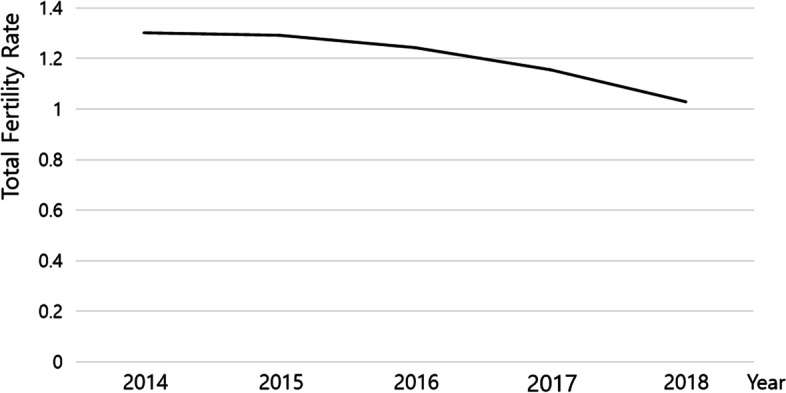
Estimation of quadratic function change model of fertility rate in South Korea

According to the analysis result of the finally selected unconditional quadratic function change model, the average of the initial fertility rate (fertility rate in 2014) was 1.316 (*p* < .001), and the linear rate of change in the Korean fertility rate was 0.016 (*p*. <0.05), and the rate of change of the quadratic function was − 0.020 (*p* < .001) (Table 3; Fig. 1). All the values were statistically significant. It means that the fertility rate in South Korea is in the form of a gradual and abrupt decline as the rate of decline increases over time. Also, the variance was significant with the initial value of 0.062 (*p* < .001), the linear rate of change 0.004 (*p* < .05), and the quadratic function change rate of 0.003 (*p* < .05). This shows that there are significant differences in the initial level and rate of change of the fertility rate between local governments.

**Table 3:** Mean and variance of initial score and rate of change of unconditional model

Variables	Mean		Variance	
	**Estimate**	**S.E.**	**Estimate**	**S.E.**
Initial Score	1.316***	0.017	0.062***	0.006
Linear Change Rate	0.016*	0.007	0.004*	0.002
Quadratic Change Rate	− 0.020***	0.002	0.003*	0.001

*p < .05, ***p < .001

### Analysis of conditional model

In the conditional model analysis, the effect of the fertility promotion policy on the initial value and change rate of the fertility rate was examined. As a result of conditional model fit analysis, it was found that there was no problem in model analysis with χ2 = 111.122 (*p* < .001), CFI = 0.952, TLI = 0.906, and RMSEA = 0.089.

As a result of examining how the fertility incentive policy affects the initial value and the rate of change of the fertility rate, only the in-kind policy among fertility policies has a significant effect on the initial value of the fertility rate (Coef.= 0.080, *p* < .001) (Table 4). In other words, it was found that the higher the number of in-kind policies, the higher the fertility rate. On the other hand, it was found that cash policy, voucher policy, service policy, education and promotion policy did not affect the initial value of fertility rate. In addition, it was found that all the policies did not significantly affect the linear rate of change and the rate of change of the quadratic function.

**Table 4:** Effect of fertility promotion policies on changes in fertility rate

Path between Variables			Coef.	S.E.
Cash Policy	→	Initial Fertility Rate	0.001	0.016
In-kind Policy	→	Initial Fertility Rate	0.080***	0.021
Voucher Policy	→	Initial Fertility Rate	0.030	0.035
Service Policy	→	Initial Fertility Rate	− 0.008	0.005
Education and Promotion Policy	→	Initial Fertility Rate	− 0.015	0.010
Cash Policy	→	The Rate of Linear Change in Fertility	0.001	0.006
In-kind Policy	→	The Rate of Linear Change in Fertility	− 0.005	0.008
Voucher Policy	→	The Rate of Linear Change in Fertility	0.014	0.013
Service Policy	→	The Rate of Linear Change in Fertility	− 0.001	0.004
Education and Promotion Policy	→	The Rate of Linear Change in Fertility	0.003	0.004
Cash Policy	→	The Rate of Quadratic Function Change in Fertility	0.001	0.001
In-kind Policy	→	The Rate of Quadratic Function Change in Fertility	0.001	0.002
Voucher Policy	→	The Rate of Quadratic Function Change in Fertility	− 0.003	0.003
Service Policy	→	The Rate of Quadratic Function Change in Fertility	0.000	0.001
Education and Promotion Policy	→	The Rate of Quadratic Function Change in Fertility	0.000	0.001

***p < .001

## Discussion

During the ultra-low fertility crisis facing the world, South Korea is currently unable to escape from the lowest fertility rate. Since the mid-2000s, the Republic of South Korea has established comprehensive government-level countermeasures against low fertility and mobilized enormous financial resources to implement various policies to encourage childbirth. However, the fertility rate is currently the only OECD member country with a total fertility rate of zero rather than rebounding [35]. Thus, this study tried to review the present state of South Korea by examining the relationship between the changes in the fertility rate in South Korea and the fertility policies implemented by local governments from a longitudinal perspective. To this end, the relationship between the number of fertility policies from 2014 to 2018 and the total fertility rate was analyzed by applying the Latent Growth Curve Model, using the casebook of fertility policies published annually by the Ministry of Health and Welfare and the total fertility rate of Statistics Korea.

As a result of the analysis, the change in the fertility rate in South Korea shows changes over time, and the shape of the trajectory is close to a curved trajectory in the form of a quadratic function. In other words, as time goes by, the rate of decline increases, indicating that the problem of declining fertility rates is very serious.

In addition, it was found that the fertility promotion policies proposed by South Korea did not have a positive effect on the fertility rate and did not slow the rate of decline in the fertility rate. South Korea has been implementing related policies until now through the establishment of the Basic Plan for Low Fertility and Aging Society in 2006, and the number of related policies, which was 1,306 in 2014, at the time of this study, reached 1,747 in 2018 [36, 37]. Based on the national budget, the amount of fiscal input to counter the low birth rate has continuously increased from KRW 1.0 trillion in 2006 to KRW 42.9 trillion in 2021 [28]. Likewise, the number and budget of fertility-related policies in South Korea have continued to increase. However, the results of this study show that these various policies do not have a positive effect on the fertility rate in the short or long term. This shows that a large-scale reorganization and overhaul of the fertility promotion policy is necessary, and at the same time, it shows that the effect of cash support, which has been overheated in the form of simply increasing the number of related policies or regional competition, is no longer effective. It is also a result of showing that current policies do not reflect regional characteristics.

On the other hand, it was found that the in-kind policy had a temporary effect on the fertility rate. In the case of in-kind policies, it is mainly implemented during pregnancy and childbirth. Typically, nutritional supplements (iron supplements, folic acid supplements) are provided during the pregnancy phase, and after childbirth, childbirth celebration supplies that can help with the provision of early childbirth supplies are in progress. Compared to other policies that provide different types of support according to the order of births, it can be inferred that the results are derived from the fact that there are many contents that correspond to the support criteria for pregnant and childbirth families. For the lasting effect of this spot policy, a customized approach seems to be necessary considering more diverse life cycles.

However, it is unreasonable to conclude this study as a failure of South Korea’s fertility policy. It is because even if the effect of a policy is positive, it can be offset by other factors [24]. In addition, it may be challenging to make an immediate turnaround due to the seriousness of the low fertility rate in South Korea, which has fallen into the low fertility trap. Nevertheless, the rapid decline in the fertility rate suggests that the decision-making process for childbirth is too complicated to be solved with only cash support or childcare services, which account for a significant portion of the childbirth promotion policy. In other words, it suggests the need to reconsider the entire government’s fertility support policy.

Several studies examining the effectiveness of South Korea’s fertility policies have determined that related policies have a positive effect on marital fertility but have a negative correlation with the marriage rates of single individuals. Pointing out that factors may differ, it is impossible to say that the policy to encourage childbirth is ineffective [38]. In addition, there are reports that the effects of policies work differently depending on family characteristics (e.g., number of children) [39, 40]. Expansion of policy at the fertility level does not help improve the fertility rate, and it shows the limitations of South Korea’s fertility policy, which focuses on childbirth. It may be the time to break away from the married person-centered policies and discuss impactful policies by considering measures to reduce single or late marriage or to reflect the diversity of families.

In this regard, this study has obvious limitations. This study looked at quantitatively by measuring only numbers without considering the characteristics of each policy. A broader discussion will be possible if we consider the qualitative aspects of the policy, such as considering the various characteristics of the policy and examining the total fertility rate in detail. Nevertheless, this study is meaningful in that it explores the relationship by examining changes in the fertility rate and policy factors at a quantitative level.

## Conclusion

In this study, we examined the changes in ts'he Korean fertility rate and the relationship between changes in the fertility rate and policies to encourage fertility. Consequently, the change in the fertility rate in South Korea showed a rapid increase in the rate of decline over time, and it was found that the fertility incentive policy did not affect both the fertility rate and the rate at which the fertility rate decreased. It shows that the quantitative expansion of the fertility promotion policy, which has been overheated in local governments, does not have a positive effect on the fertility rate in the short and long term and does not reflect the region’s characteristics. For the future studies, an impactful policy with a more precise purpose and target is necessary rather than quantitative expansion.

## Data Availability

The data that support the findings of this study are available from the corresponding author upon reasonable request.
